# Thoracic Myelopathy Associated with Persistent Idiopathic Scoliosis in Adulthood: A Case Report

**DOI:** 10.7759/cureus.97967

**Published:** 2025-11-27

**Authors:** Shunsuke Iwai, Satoshi Suzuki, Kazuki Takeda, Kota Watanabe, Masaya Nakamura

**Affiliations:** 1 Department of Orthopaedic Surgery, Keio University School of Medicine, Tokyo, JPN

**Keywords:** adulthood, double-major curves, idiopathic scoliosis, spinal instability, thoracic myelopathy

## Abstract

We report a rare case of thoracic myelopathy associated with persistent idiopathic scoliosis in adulthood. Despite having a thoracic Cobb angle of more than 50°, the patient refused corrective surgery after being diagnosed with idiopathic scoliosis at age 16. Even in adulthood, the scoliotic curves continued to progress, leading to instability at T11/12 and spinal canal stenosis with intramedullary signal change. This case highlights that long-standing severe thoracic and thoracolumbar double-major curves can lead to instability at the thoracolumbar junction, ultimately resulting in neurological deficits.

## Introduction

Adolescent idiopathic scoliosis (AIS) is a three-dimensional spinal deformity that occurs in approximately 1-3% of adolescents [1]. Surgery should be considered when the Cobb angle of the primary curve is greater than 40° to 50°; however, some patients do not wish to undergo surgery even if the Cobb angle is indicated for surgery [2]. Although untreated AIS is known to progress slightly in adulthood, increased spinal deformity of AIS rarely leads to neurological deficits [3]. Here, we report a rare case of thoracic myelopathy associated with persistent idiopathic scoliosis in adulthood that was successfully treated via surgery. Although thoracic myelopathy secondary to degenerative scoliosis has been described, similar reports arising from long-standing, untreated AIS are lacking [4]. The purpose of this report is to clarify how untreated, rigid double-major curves can progress to degenerative instability that ultimately results in thoracic cord compression, and to outline the associated surgical decision-making.

## Case presentation

A 62-year-old man with a 1-year history of progressive numbness in bilateral lower limbs, gait disturbance, and dysuria was referred to our hospital. He was previously diagnosed with idiopathic scoliosis at the age of 16 years, and his doctor recommended corrective surgery because the Cobb angle of the thoracic curve exceeded 50°; however, the patient opted for continuous observation. He also had a history of hypertension, asthma, and reflux esophagitis; however, none of these systemic comorbidities were considered to have influenced the progression of the spinal deformity.

The patient had a spastic gait and was unable to walk without any assistance. Physical examination showed that the muscle strength was normal, but sensory disturbance below the waistline and hyperreflexia in both lower extremities were observed. Pathological tendon reflexes, including Babinski reflexes and ankle clonus, were positive in both legs. The Japanese Orthopedic Association (JOA) score for thoracic myelopathy was 5.5 (2, 2, 0.5, and 1) out of 11 points [5,6].

**Figure 1:**
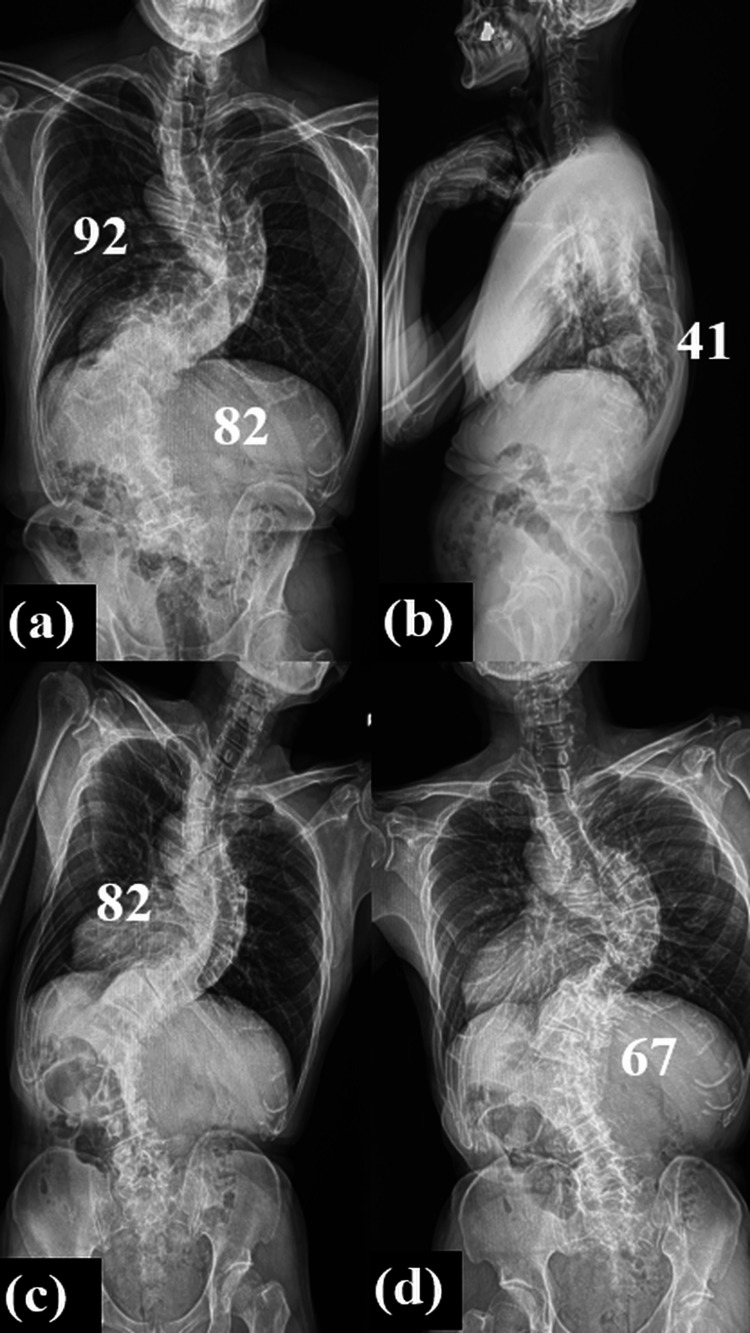
Preoperative radiographs Preoperative (a, b) standing posteroanterior radiographs showing the Cobb angles of the (a) thoracic and lumbar curves (92° and 82°, respectively) and (b) thoracolumbar kyphosis (41°) and (c, d) side-bending radiographs showing loss of flexibility.

Standing whole-spine radiographs revealed scoliosis with a thoracic Cobb angle of 92° at T5-T11 and a lumbar Cobb angle of 87° at L11-L4 (Figure 1a). There was local kyphosis of 41° at the thoracolumbar transition region, but global balance was maintained (Figure 1b). On side-bending radiographs, the thoracic curve showed only minimal correction from 92° to 82°, and the lumbar curve corrected from 82° to 67°, consistent with a rigid deformity (Figure 1c, 1d). Each parameter is shown in Table 1. The patient was diagnosed with SRS-Schwab classification type D (0, +, +).

**Table 1:** Radiographic parameters of the patient CSVL: central sacrum vertical line; PI: pelvic incidence; PT: pelvic tilt; LL: lumbar lordosis; TK: thoracic kyphosis; SVA: sagittal vertical axis.

	Preoperative	Final follow-up
Cobb angle of the thoracic curve (T5-11) (°)	92	60
Cobb angle of the lumbar curve (T11-L4) (°)	82	50
CSVL (mm)	50	6
Local kyphosis (T10-L2) (°)	41	19
PI (°)	46	41
PT (°)	28	10
LL (°)	40	40
TK (T5-12) (°)	30	33
PI-LL mismatch (°)	6	1
SVA (mm)	31	15

**Figure 2:**
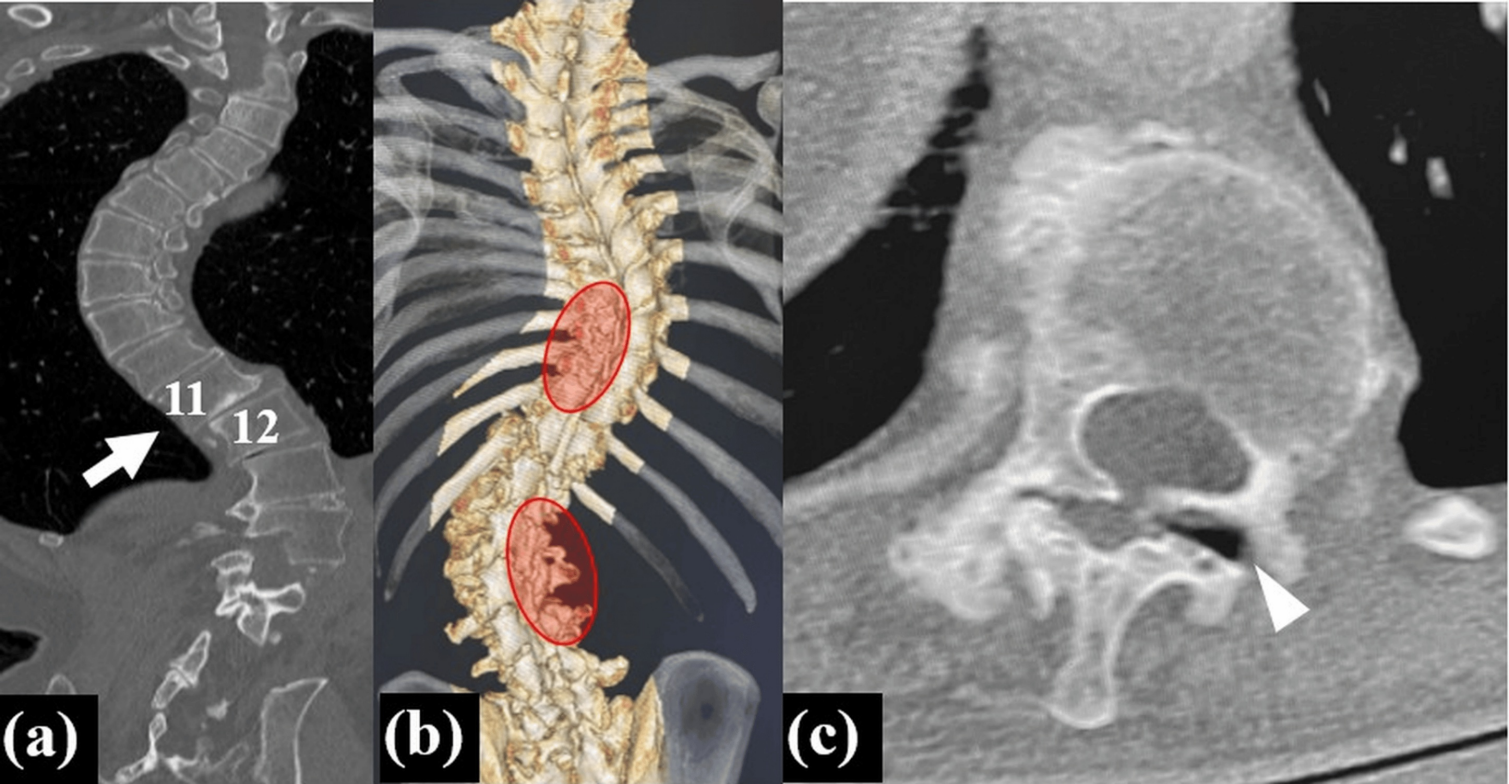
Preoperative computed tomography (CT) (a) Coronal CT showing lateral slip between T11 and T12 (white arrow). (b) Three-dimensional CT demonstrating concave-side facet fusion between T8-11 and L1-4. (c) Axial CT showing bilateral facet osteoarthritis and left T11/12 facet opening (white arrowhead).

**Figure 3:**
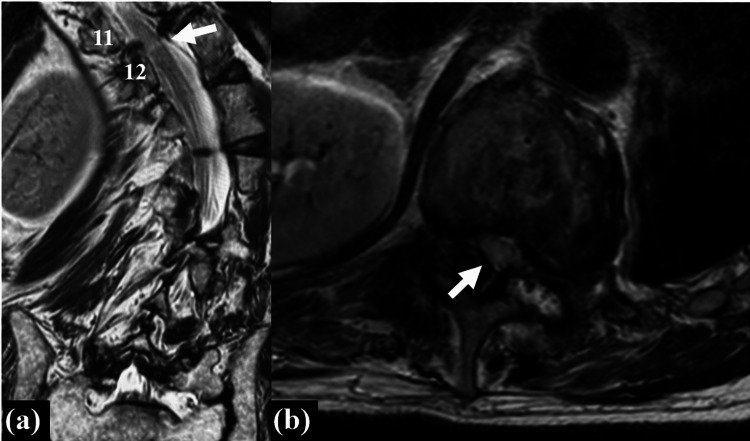
Preoperative T2-weighted magnetic resonance imaging (MRI) (a) (b) T2-weighted MRI showing stenosis with high intramedullary signal at T11–12 (white arrows).

Computed tomography (CT) revealed a lateral slip of T11 vertebra (Figure 2a). Three-dimensional CT revealed facet fusion of the concave sides between T8-11 and T12-L3 (Figure 2b). Osteoarthritic change between T11/12 and the facet opening of left T11/12 was observed, indicating severe spinal instability between T11 and T12 (Figure 2a, 2c). T2-weighted magnetic resonance imaging revealed intramedullary signal change at the T11/12 level (Figure 3a,b).

**Figure 4:**
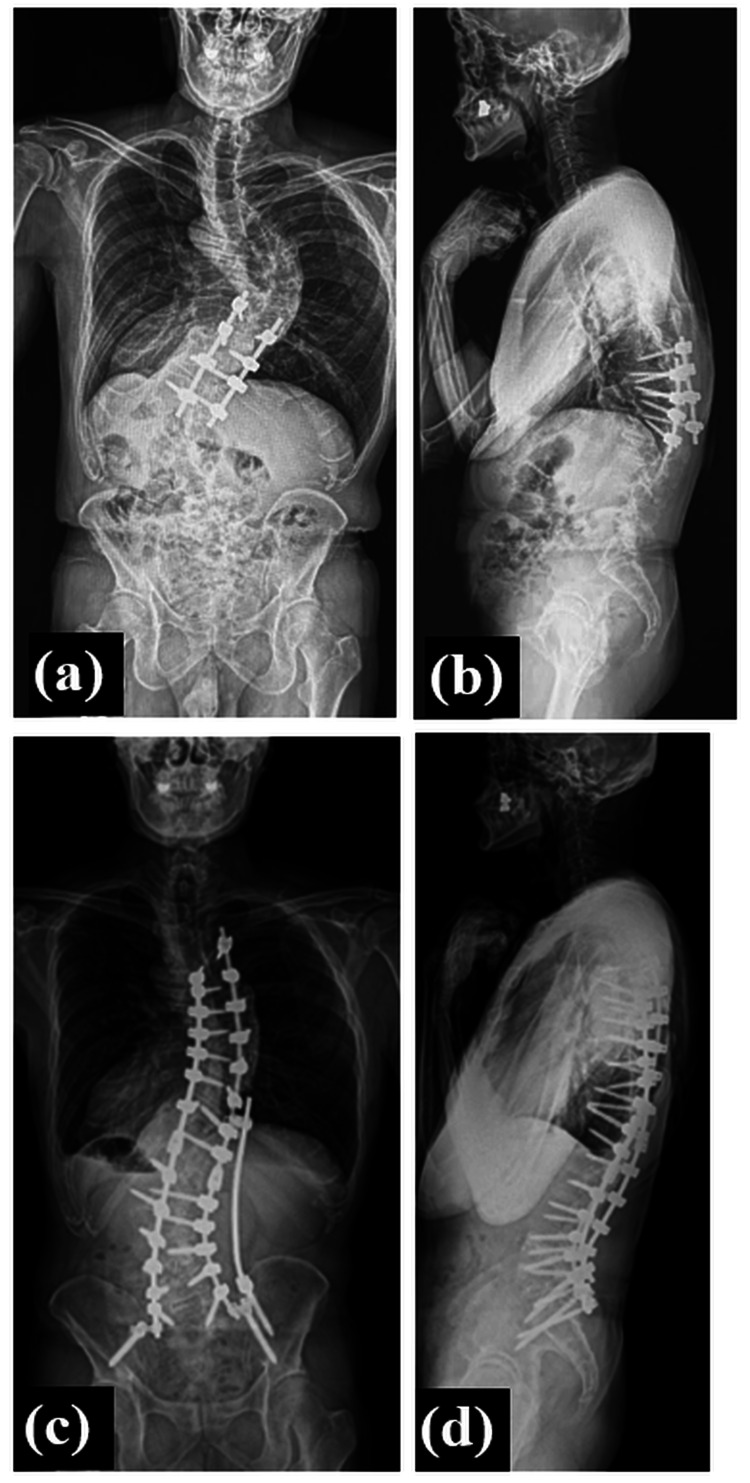
Postoperative radiographs Postoperative standing posteroanterior radiographs obtained one year after the first surgery (a, b) and at the final follow-up after the second surgery (c, d).

**Figure 5:**
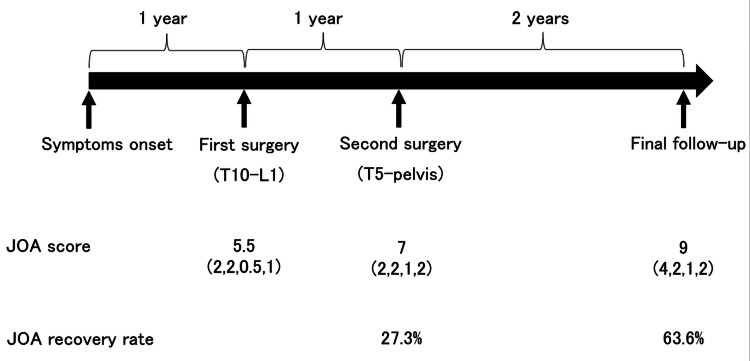
Timeline of the patient

The patient was diagnosed with progressive thoracic myelopathy caused by progressive idiopathic scoliosis in adulthood. As the spinal symptoms and bladder and rectal disorders were progressing, we performed posterior decompression and fusion from T10 to L1 (2 above-2 below) (Figure 4a, 4b). Although the patient’s myelopathic symptoms improved, posterior correction and fusion from T5 to the pelvis were additionally performed one year after the first surgery due to residual low back pain and gait disturbance associated with spinal deformity (Figure 4c, 4d). At the last follow-up, two years after the revision surgery, the JOA score improved to 9 (4, 2, 1, and 2) points (recovery rate: 63.6%)(Figure 5).

## Discussion

Thoracic myelopathy is a relatively uncommon degenerative disease compared with degenerative pathology in the cervical and lumbar spines [7]. Limited range of motion and the stability of the rib cage could contribute to the lower frequency. Degenerative changes, such as ossification of the posterior longitudinal ligament (OPLL), ossification of the ligamentum flavum (OLF), thoracic disc herniation, tumor, infection, and vertebral fracture, are the possible pathologies of thoracic myelopathy. In a report of 300 cases of thoracic myelopathy conducted by Ando et al., the most common cause was intradural extramedullary tumor (n = 98), followed by intramedullary spinal cord tumor (n = 64), OLF (n = 48), OPLL (n = 30), OPLL with OLF (n = 27), vertebral tumor (n = 17), spinal cord herniation (n = 7), thoracic disc herniation (n = 5), and vertebral fracture (n = 4) [8]. No description concerning scoliosis was presented in the reports. Pettigrew et al. conducted a cadaveric study and showed that the main thoracic scoliotic deformity did not significantly increase thoracic spinal cord intramedullary pressure [9]. Based on these results, scoliosis was considered to be an extremely rare cause of thoracic myelopathy.

Many studies reported on thoracic myelopathy or paraplegia due to mechanical compression and traction by sharply angular kyphoscoliosis and intraspinal rib head displacement related to neurofibromatosis 1 [10-12]. Proximal thoracic kyphoscoliosis related to congenital scoliosis has also been reported as a cause of thoracic myelopathy [13-15]. The prevalence rates of myelopathy in congenital kyphoscoliosis were reported to be 9.8% to 12.3% [13,14]. Matsumoto et al. reported the case of a 14-year-old adolescent boy with congenital kyphoscoliosis with T5 hemivertebra and concluded that myelopathy could occur even if the kyphosis angle was small (43°) [15]. Most cases of syndromic and congenital scoliosis/kyphoscoliosis with myelopathy occur at the apex of the deformity during a growth spurt in adolescents. Kamiya et al. reported a case of a 56-year-old man with not only neuromuscular scoliosis but also diffuse idiopathic skeletal hyperostosis who presented with gait disturbance [16]. Severe stenosis was observed at the T11/12 level, and the patient was successfully treated via decompression with 1 above-1 below fixation surgery. Although the pathology of their patient was similar to that of our patient, to our knowledge, ours is the first reported case of thoracic myelopathy in a patient with idiopathic scoliosis in adulthood.

Many possible reasons for thoracic myelopathy occurring at the T11/12 level in our case were speculated upon. In general, T11/12 is not enclosed by the rib cage, and degenerative changes are prone to occur in the thoracolumbar area. Furthermore, T11/12 is the inflection area of the structural thoracic and thoracolumbar/lumbar curvature, resulting in the concentration of mechanical stress between T11 and T12. One of the major pathologies of thoracic myelopathy was the lateral slip of T11. Kotani et al. reported that older age, especially >37 years, is a risk factor for lateral slip in patients with residual AIS [17]. They also reported that lateral slip was most common at L3-4; However, they did not report lateral slip of the thoracic vertebrae. Another speculated pathology is instability between T11 and T12 owing to the loss of flexibility between the double major structural curvatures. Residual severe curvature in adulthood results in degenerative changes of the T11/12 facet joint, left T11/12 facet opening, and spontaneous facet fusion of both concave sides. Hypokyphosis in scoliosis has been reported to be associated with long-term progressive degeneration of the lower thoracic intervertebral discs, suggesting that reduced kyphosis of the main thoracic curve may have promoted degenerative changes at the thoracolumbar junction [18]. All these factors might induce concentrated mechanical stress between T11 and T12, leading to thoracic myelopathy at T11/12. This case highlights the importance of recognizing that long-standing, untreated, and rigid AIS may progressively develop secondary degenerative instability, leading to neurological compromise, similar to adult de novo scoliosis. Careful monitoring, including periodic MRI surveillance, may be warranted in symptomatic chronic untreated cases.

## Conclusions

We describe a rare case of thoracic myelopathy in an adult with persistent idiopathic scoliosis. This case highlights that severe, long-standing rigid scoliosis, especially thoracic and thoracolumbar double-major curves, can lead to neurological compromise in adulthood, underscoring the need for careful monitoring and ongoing surveillance for subtle or progressive neurologic signs.
